# Sub-Analysis of CYP-GUIDES Data: Assessing the Prevalence and Impact of Drug-Gene Interactions in an Ethnically Diverse Cohort of Depressed Individuals

**DOI:** 10.3389/fphar.2022.884213

**Published:** 2022-04-12

**Authors:** Rustin D. Crutchley, Nicole Keuler

**Affiliations:** ^1^ Department of Pharmacotherapy, College of Pharmacy and Pharmaceutical Sciences, Washington State University, Yakima, WA, United States; ^2^ School of Pharmacy, University of the Western Cape, Cape Town, South Africa

**Keywords:** ethnicity, race, length of stay, drug-gene interactions, depression, CYP2D6 phenotype

## Abstract

**Introduction:** Minority groups are underrepresented in pharmacogenomics (PGx) research. Recent sub-analysis of CYP-GUIDES showed reduced length of stay (LOS) in depressed patients with CYP2D6 sub-functional status. Our primary objective was to determine whether PGx guided (G) versus standard treatment (S) influenced LOS among different race/ethnic groups. Secondary objectives included prevalence of drug-gene interactions (DGIs) and readmission rates (RAR).

**Methods:** Retrospective sub-analysis of CYP-GUIDES data comprising CYP2D6 phenotypes was reclassified using standardized CYP2D6 genotype to phenotype recommendations from the Clinical Pharmacogenetics Implementation Consortium (CPIC) and Dutch Pharmacogenetics Working Group (DPWG). The Mann-Whitney test was used to determine differences in LOS between groups G and S and Kruskal Wallis test to compare LOS among different race/ethnic groups. Logistic regression was used to determine covariates associated with RAR.

**Results:** This study included 1,459 patients with 67.3% in G group (*n* = 982). The majority of patients were White (57.5%), followed by Latinos (25.6%) and Blacks (12.3%). Although there were no differences in LOS between G and S groups, Latinos had significant shorter LOS than Whites (*p* = 0.002). LOS was significantly reduced by 5.6 days in poor metabolizers in group G compared to S (*p* = 0.002). The proportion of supra functional and ultra-rapid metabolizers (UMs) were 6 and 20.3% using CYP-GUIDES and CPIC/DPWG definitions, respectively. Prevalence of DGIs was 40% with significantly fewer DGIs in Blacks (*p* < 0.001). Race/ethnicity was significantly associated with RAR (aOR 1.30; *p* = 0.003).

**Conclusion:** A greater number of patients were classified as CYP2D6 UMs using CPIC/DPWG definitions as compared to CYP-GUIDES definitions. This finding may have clinical implications for using psychotropics metabolized by CYP2D6.

## Introduction

Depression is the leading cause of disability worldwide with 16 million Americans suffering from moderate or severe depression and costs exceeding $210 billion annually (Vos et al., 2012; Bradley et al., 2018). Antidepressants are commonly prescribed in the United States (US) with approximately 10% of adults reporting recent use in the past month (Bradley et al., 2018). Despite common antidepressant use, only one-third of newly diagnosed depressed individuals achieve remission during their first treatment course (Trivedi et al., 2006; Gaynes et al., 2009). Reasons for treatment failure include baseline severity/duration of depression, underlying neuropathology, concurrent medical conditions, and poor tolerability. This is significant because consequences of ineffective treatment of depression lead to a worsening disease course, higher suicidal risk, and greater societal and economic burden.

Current treatment approaches for depression are insufficient relying on a trial-and-error approach. Drug-gene interactions (DGIs) play a contributory role in inter-patient variability in treatment response to antidepressants. These can manifest as different drug metabolizer status phenotypes [i.e., poor metabolizer (PM)/ultra-rapid metabolizer (UM)] and/or ineffective drug target signaling involving serotonin transporters and receptors (Hicks et al., 2015; Caudle et al., 2017). Integration of pharmacogenomics (PGx) into healthcare practices (i.e., clinical practice, clinical trials and pharmacovigilance programs) (Sosa-Macías et al., 2016), can lead to a personalized medicine approach, ultimately improving patient response and assisting patients to reach targeted treatment outcomes promptly. The latter is significant since early response to therapy in depressed individuals is a predictor of treatment outcome (Beard and Delgadillo, 2019). Knowledge of PGx for a patient can help guide the prescriber in identifying the most appropriate medication before initiating therapy, reduce adverse events (Zhou et al., 2015) and hospital admission, and promote medication safety (Panza et al., 2016). Recent clinical studies involving treatment resistant depressed patients have reported significantly improved remission rates for those whose DGIs were taken into consideration versus those receiving treatment as usual (Hall-Flavin et al., 2012, 2013; Winner et al., 2013; Singh, 2015; Pérez et al., 2017; Bradley et al., 2018; Greden et al., 2019; Thase et al., 2019).

Although combinatorial panel-based PGx testing has improved medication selection and cost effectiveness for those with underlying DGIs, the generalizability of these findings to different race/ethnic populations remains unknown because of their large under-representation in clinical studies (Rush et al., 2006; Lesser et al., 2007; Claudio-Campos et al., 2015). For example, according to the genome-wide association study (GWAS) catalog, although individuals of European descent comprise only 16% of the world population, almost 80% are included as all GWAS participants (Martin et al., 2019). In 2020, White non-Hispanics were the most prevalent ethnic group (57.8%) in the United States followed by Latinos accounting for the second largest group (18.7%) and then by Blacks/African Americans (12.1%) (Jensen et al., 2021). Race/ethnicity is an important consideration since the prevalence of genetic variants in drug metabolism may vary across different race/ethnic groups and could ultimately influence therapeutic recommendations (Shah and Gaedigk, 2018). Although it is unclear what threshold constitutes race/ethnic diversity, Federal agencies such as the National Human Genomic Research Institute encourage inclusion and recruitment of under-represented minorities into genomics-related research (Bonham and Green, 2021).

Length of stay (LOS) during hospitalization can be influenced by CYP2D6 phenotype. Impaired functional status in CYP2D6 has been associated with prolonged LOS while UM CYP2D6 phenotype has been associated with increased risk for readmission rates (RAR) (Laika et al., 2009; Ruaño et al., 2013; Takahashi et al., 2017). Both LOS and RAR (Takahashi et al., 2017) influence the financial cost of patients living with major depressive disorder (MDD) and increase the disease burden (Jencks et al., 2009).

The CYP-GUIDES (Cytochrome Psychotropic Genotyping Under Investigation for Decision Support) randomized clinical trial included patients with MDD admitted to one location at the Institute of Living at Hartford Hospital (Ruaño et al., 2020). A total of 1,500 depressed individuals were randomized (2:1) to Genetically guided therapy (Group G, *n* = 982) and Standard Care (Group S, *n* = 477). This original, prospective, randomized controlled trial reported no differences in 1,459 depressed, hospitalized patients for LOS and RAR between the CYP2D6 genetically-guided and standard therapy groups (Ruaño et al*.*, 2020). One of the strengths of this study is that it included a racially and ethnically diverse population as it included Blacks and Latinos. In this study, Latinos had a significantly shorter LOS than Whites. Authors of this study suggested that potential confounders such as these could have obscured the results of using PGx guidance for treatment of depression. A recent subgroup analysis of this study concluded that PGx guided therapy reduced the LOS in depressed patients with sub-functional CYP2D6 status who were prescribed CYP2D6 major psychotropic medications (Ruaño et al*.*, 2021). This sub-analysis specifically addressed three potential confounders such as a single electronic medical record, a minimum 3-day LOS, and stratification of patients by CYP2D6 phenotype.

Data analyses from both the CYP-GUIDES trial and the subsequent sub-analysis excluded patients who were supra-functional CYP2D6 metabolizers because of their lower representation (6.2 and 6.7%, respectively) of the total cohort that could lead to underpowered data comparisons, especially, with respect to treatment outcomes involving LOS and RAR. As noted by the authors in these studies, functional categories for CYP2D6 were implemented in 2014. However, recent consensus recommendations from Clinical Pharmacogenetics Implementation Consortium (CPIC) and the Dutch Pharmacogenetics Working Group (DPWG) were published regarding standardization of CYP2D6 genotype to phenotype (Caudle et al., 2020). These recommendations support a UM in CYP2D6 as having a total activity score of greater than 2.25.

Given the need for including underrepresented groups in clinical PGx studies and the recent updated standardization of CYP2D6 phenotypes, the primary objective of our analysis was to determine whether pharmacogenetics-guided (Group G) versus standard therapy (Group S) influenced LOS among different race/ethnicities in the CYP-GUIDES trial. Secondary objectives included determination of the prevalence of DGIs and RAR among different race/ethnic groups.

## Methods

A retrospective sub-analysis was performed using the publicly available CYP-GUIDES dataset (Tortora et al., 2020). Detailed methodology for the trial including eligibility criteria has been described previously (Ruaño et al., 2020). The dataset included anonymized data from 1,500 patients; randomized (2:1) to Genetically guided therapy (Group G, *n* = 982) and Standard Care (Group S, *n* = 477).

Two electronic medical records (EMRs) were used during the study period: the Clinical Evaluation and Monitoring System (CEMS) and Epic EMR. Study patients with no genomic information were excluded from our sub-analysis (*n* = 41). In terms of CYP2D6 genotyping, 21 common allelic variants comprising of either null, deficient or rapid function were interrogated in the CYP-GUIDES trial (Ruaño et al., 2020). The metabolic reserve (MR) index incorporated in the CYP-GUIDES trial was used to quantify CYP450 functional phenotypes (Ruaño et al., 2011; Villagra et al., 2011). The MR index equivalent to a total activity score is calculated by adding the activity score of each of the two CYP2D6 alleles for each patient (Tortora et al., 2020). These phenotypes included the following categories: sub-functional (activity score (AS ≤ 1.0) or MR of 0.0, 0.5 or 1.0, functional (1.5 ≤ AS<2.5) or MR of 1.5, 2.0 or 2.5, and supra-functional (AS ≥ 3.0) or MR of 3.0 or 3.5. In our sub-analysis, phenotypes for patients were reclassified using updated standardization of CYP2D6 genotype to phenotype translation from consensus recommendations from CPIC and the DPWG (Caudle et al., 2020). These latter phenotypes included the following: PMs with AS = 0; intermediate metabolizers (IMs) ranging 0 < AS < 1.25; normal metabolizers (NMs) ranging 1.25 ≤ AS ≤ 2.25; and UMs with an AS > 2.25.

LOS was defined as the duration of inpatient care; time from admission till discharge (Segen’s Medical Dictionary, 2002). RAR was defined as hospitalization within 30 days of discharge of the current admission (Ruaño et al*.*, 2020). Diagnosis was stratified by six categories: depression, MDD without psychotic features, MDD with psychotic features, MDD recurrent, MDD recurrent with psychotic features and other. In our sub-analysis, a DGI was defined as follows: a patient who is either a PM (AS of zero), IM (AS of 0.5 and 1), or UM (AS of 2.5, 3 and 3.5) and is administered a major CYP2D6 substrate psychotropic medication at least once during hospitalization.

We restricted some of our data analyses to patients who had a LOS >3 days to account for the turn-around time necessary for physicians to obtain CYP2D6 genotyping results for therapeutic guidance. Additional data analyses were conducted where necessary by including only CEMS, since this was the more familiar EMR health care providers had been using before transitioning to the Epic EMR during the clinical trial.

The Mann-Whitney test was used to determine differences in LOS between groups G and S. The Kruskal Wallis test was used to determine differences in LOS among different race/ethnic groups with the Dunn test with Bonferroni adjustment as a post hoc test (adjusted alpha = 0.013). Analysis of variance (ANOVA) was used to determine differences in LOS between race/ethnicities in groups G and S. Logistic regression was used to determine potential covariates or confounders associated with RAR and DGIs. All data analyses were conducted with Stata 17 (Stata Corp LCC, College Station, TX).

## Results

### Demographics and Prevalence of Drug-Gene Interactions (DGIs)

This study included 1,459 patients with 67.3% in group G (*n* = 982). Majority of patients were White (57.5%), followed by Latinos (25.6%) and Blacks (12.3%). MDD with recurrence was the most prevalent reported diagnosis (42.2%), with a higher prevalence in group G (43.3%) compared to S (39.8%). Whites reported more MDD with recurrence (*n* = 399, 47.6%) compared to Blacks (*n* = 71, 40%) and Latinos (*n* = 113, 30.3%). MDD with recurrence with psychotic features was more prevalent among Latinos (*n* = 42, 11.3%) compared to Blacks (*n* = 9, 5%) and Whites (*n* = 28, 3.3%). Similarly, MDD with psychotic features was most prevalent among Latinos (*n* = 61, 16.4%) compared to Blacks (*n* = 17, 9.5%) and Whites (*n* = 29, 3.5%).

DGIs were comparable between Groups G (39.5%) and S (40%) with an overall prevalence of 39.7% in the study. Significantly fewer DGIs were present among Blacks compared to Latinos (24 versus 42.9%, respectively; OR = 2.3 95% CI 1.6-3.5, *p* < 0.001) and Whites (24 versus 42.2%, respectively; OR 2.2 95% CI 1.5-3.2, *p* < 0.001). However no significant differences were observed between DGIs in group G and S (*p* = 0.7) as well as between other demographic variables in groups G and S. Refer to Table 1 for baseline demographics of the study population.

**TABLE 1:** Demographics of study population.

	Group G *n* (%)	Group S *n* (%)	Total *n* (%)
Total study population N (%)	982 (67.31)	477 (32.69)	1,459 (100)
Female (%)	504 (51.3)	240 (50.3)	744 (51.0%)
Age, median [IQR]	37 [24–51]	37 [25–51]	37 [38.7]
Race/ethnicity			
Latino	249 (25.4)	124 (26.0)	373 (25.6)
Black	111 (11.3)	68 (14.3)	179 (12.3)
White	578 (58.9)	260 (54.5)	838 (57.4)
Unknown	44 (4.5)	25 (5.2)	69 (4.7)
Electronic record			
Clinical Evaluation and Monitoring (CEMS)	549 (55.9)	277 (58.1)	826 (56.6)
Epic	433 (44.1)	200 (41.9)	633 (43.4)
Diagnosis at admission			
Depression	172 (17.5)	97 (20.3)	269 (18.4)
MDD without psychotic features	252 (25.7)	123 (25.8)	375 (25.7)
MDD with psychotic features	77 (7.8)	35 (7.3)	112 (7.7)
MDD recurrent	426 (43.4)	190 (39.8)	616 (42.2)
MDD recurrent with psychotic features	52 (5.3)	30 (6.3)	82 (5.62)
Other	3 (0.3)	2 (0.4)	5 (0.3)
Number of patients prescribed a major substrate CYP2D6 psychotropic medication	633 (64.5)	321 (67.3)	954 (65.4)
Latino	167 (67.0)	92 (74.2)	259 (69.4)
Black	60 (54.1)	42 (61.8)	102 (57.0)
White	381 (65.9)	172 (66.2)	553 (66.0)
Drug-gene interactions (DGIs)^a^	388 (39.5)	191 (40.0)	579 (39.7)
Latino	105 (42.2)	55 (44.4)	160 (42.9)
Black	24 (21.6)	19 (27.9)	43 (24.0)^b^
White	245 (42.4)	109 (41.9)	354 (42.2)
Total number of psychotropics prescribed, median [IQR]	2 [2–4]	3 [2–3]	3 [2–4]
Latino	3 [2–4]	3 [2–3]	3 [2–4]
Black	2 [1–3]	2 [2–3]	2 [2–3]
White	2 [2–4]	3 [2–4]	3 [2–4]

a In our sub-analysis, definition of a drug-gene interaction (DGI) was defined as a patient who is either a PM (AS, of zero), IM (AS, including 0.5 and 1), or UM (includes AS, of 2.5, 3 and 3.5) and is administered a major CYP2D6 substrate psychotropic medication at least once during hospitalization.

b Overall, Blacks experienced significantly less DGIs, compared to Whites (*p* < 0.001) and Latinos (*p* < 0.001).

### Prevalence of CYP2D6 Phenotypes in Study Population

**FIGURE 1:**
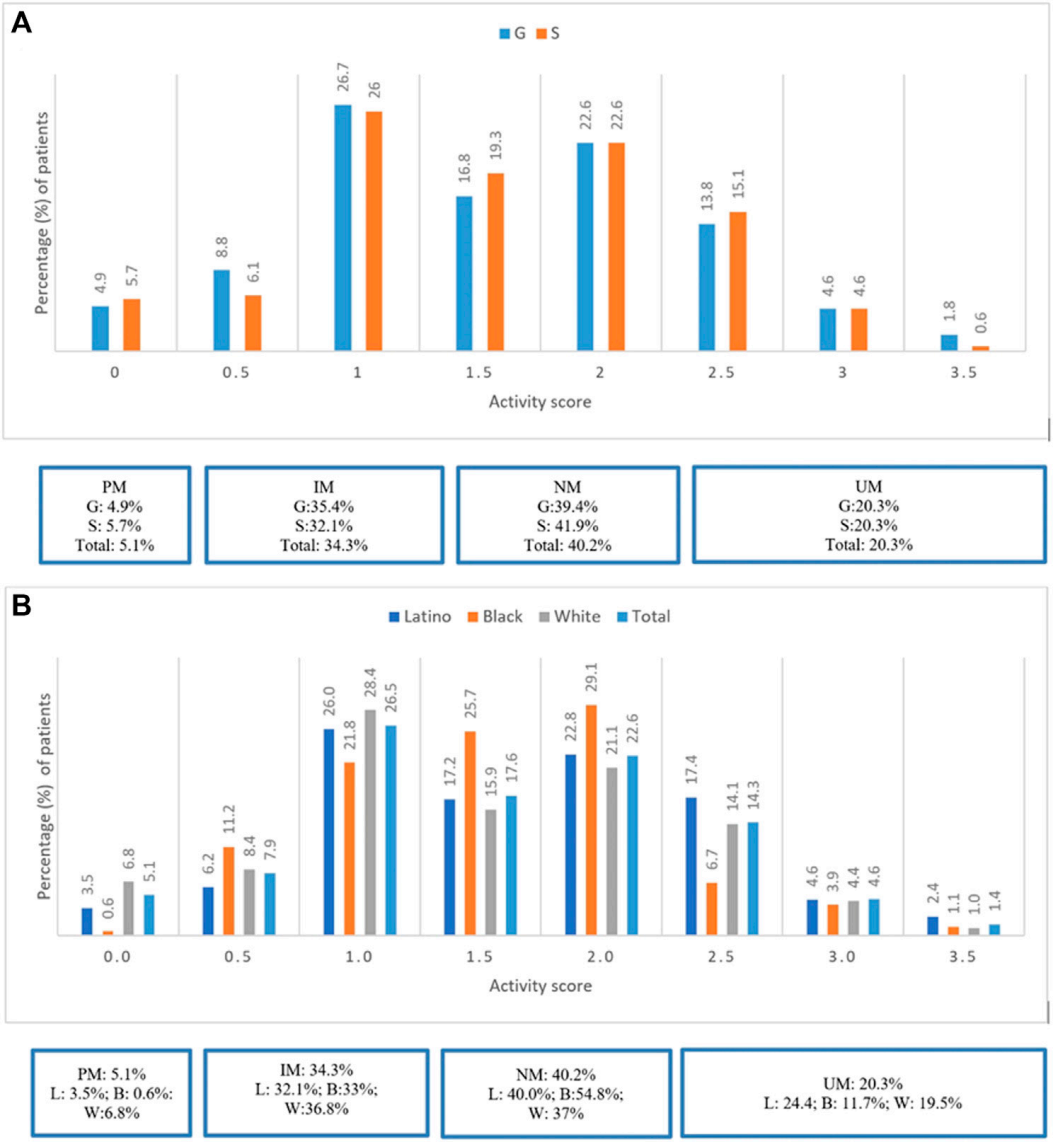
**(A)** Prevalence of CYP2D6 Phenotypes stratified by groups G and S. **(B)** Prevalence of CYP2D6 Phenotypes stratified by ethinicity for the total study population.

Most patients were CYP2D6 NMs (40.2%), followed by IMs (34.3%), UMs (20.3%), and PMs (5.1%). Refer to Figure 1A. There were no significant differences in prevalence of CYP2D6 phenotypes between groups G and S (*p* = 0.6). PMs and IMs in CYP2D6 were more prevalent among Whites (6.8 and 36.8%) compared to Latinos (3.5 and 32.1%) and Blacks (0.6 and 33%), respectively. However, UMs were more prevalent among Latinos (24.4%) compared to Whites (19.5%) and Blacks (11.7%). Refer to Figure 1B.

### Length of Stay (LOS)

The median LOS was 6.6 and 6.1 days when restricting LOS to >3 days and LOS to >3 days and using CEMS only, respectively. LOS was not significantly different between groups G and S for patients admitted for >3 days (*p* = 0.6) or admitted for >3 days using CEMS only (*p* = 0.4). Further stratification of data analysis for LOS was restricted to LOS to >3 days and using CEMS EMR only when comparing LOS between groups G and S with regards to CYP2D6 phenotype and race/ethnicity. CYP2D6 phenotype was associated with LOS (*p* = 0.03); however, only PMs in group G had a significantly shorter LOS compared to group S (median 5.8 days versus median 11.4 days; *p* = 0.002). Race/ethnicity was significantly associated with LOS (*p* = 0.004). However, no significant differences were detected in LOS between groups G and S among different race/ethnic groups including Blacks (*p* = 0.06). Overall, Latinos had a significantly shorter LOS compared to Whites (total median 5.8 versus 6.6 days, respectively; *p* = 0.002). Refer to Table 2.

**TABLE 2:** Length of Stay stratified by Groups G and S according to CYP2D6 phenotype and race/ethnicity.

	Group G Median [IQR] *n* (%)	Group S Median [IQR] *n* (%)	Total Median [IQR] *n* (%)
LOS >3 days	6.7 [5.0–8.8], 798 (67.3)	6.5 [4.7–9.5], 387 (32.7)	6.6 [4.9–8.9], 1,185 (100.0)
LOS >3 days and CEMS	6.1 [4.8–8.0], 428 (66.5)	6.0 [4.3–8.3], 216 (33.5)	6.1 [4.7–8.1], 644 (100.0)
CYP2D6 phenotype*			
Poor metabolizer	5.8 [4.8–7.5]**, 21 (4.9)	11.4 [6.2–21.1], 13 (6.0)	6.1 [5.0–9.0], 34 (5.3)
Intermediate metabolizer	6.8 [5.0–8.2], 159 (37.2)	6.0 [4.2–9.6], 75 (34.7)	6.8 [4.8–8.6], 234 (36.3)
Normal metabolizer	5.8 [4.6–7.8], 147 (34.4)	5.9 [4.0–7.5], 86 (39.8)	5.8 [4.5–7.7], 233 (36.2)
Ultra-rapid metabolizer	6.4 [5.1–8.9], 101 (23.6)	5.8 [4.4–8.0], 42 (19.4)	6.2 [4.8–8.9], 143 (22.2)
Race/ethnicity***			
Latino	5.8 [4.4–7.7], 116 (27.1)	5.6 [4.3–7.9], 60 (27.8)	5.8 [4.4–7.7], 176 (27.3)
Black	6.6 [5.1–8.9], 43 (10.1)	5.1 [4.1–6.8], 33 (15.3)	5.8 [4.5–7.8], 76 (11.8)
White	6.6 [5.0–8.5], 254 (59.4)	7.0 [4.5–11.4], 118 (54.6)	6.6 [4.9–8.8], 372 (57.8)

*p = 0.03; CYP2D6 phenotype was associated with LOS.

**p = 0.002; poor metabolizers in group G had a significantly shorter LOS, compared to group S.

***p = 0.004; race/ethnicity was significantly associated with LOS, with Latinos having a significantly shorter LOS, compared to Whites (*p* = 0.002).

### Readmission Rates (RAR)

A total of 142 patients (9.7%) were re-admitted within 30 days post discharge, with slightly more in group G (*n* = 99, 10.1%) compared to group S (*n* = 43, 9%). There were no differences in RAR for groups G and S after adjusting for confounders including age, sex, treatment group, CYP2D6 phenotype, diagnosis at admission (aOR = 0.9; *p* = 0.6). However, race/ethnicity was significantly associated with RAR (aOR = 1.3, *p* = 0.003). RAR was lower among Latinos in group G (*n* = 16, 6.4%) compared to group S (*n* = 8, 8.1%). However, for Blacks (*n* = 8, 7.2% versus *n* = 3, 4.4%) and Whites (*n* = 73, 12.6% versus *n* = 28, 10.8%), RAR was greater in group G versus group S, respectively. Overall, RAR was highest in Whites (*n* = 101, 12.1%) compared to Blacks (*n* = 11, 6.2%) and Latinos (*n* = 26, 7.0%).

### Number of Psychotropic Medication Administrations

**FIGURE 2:**
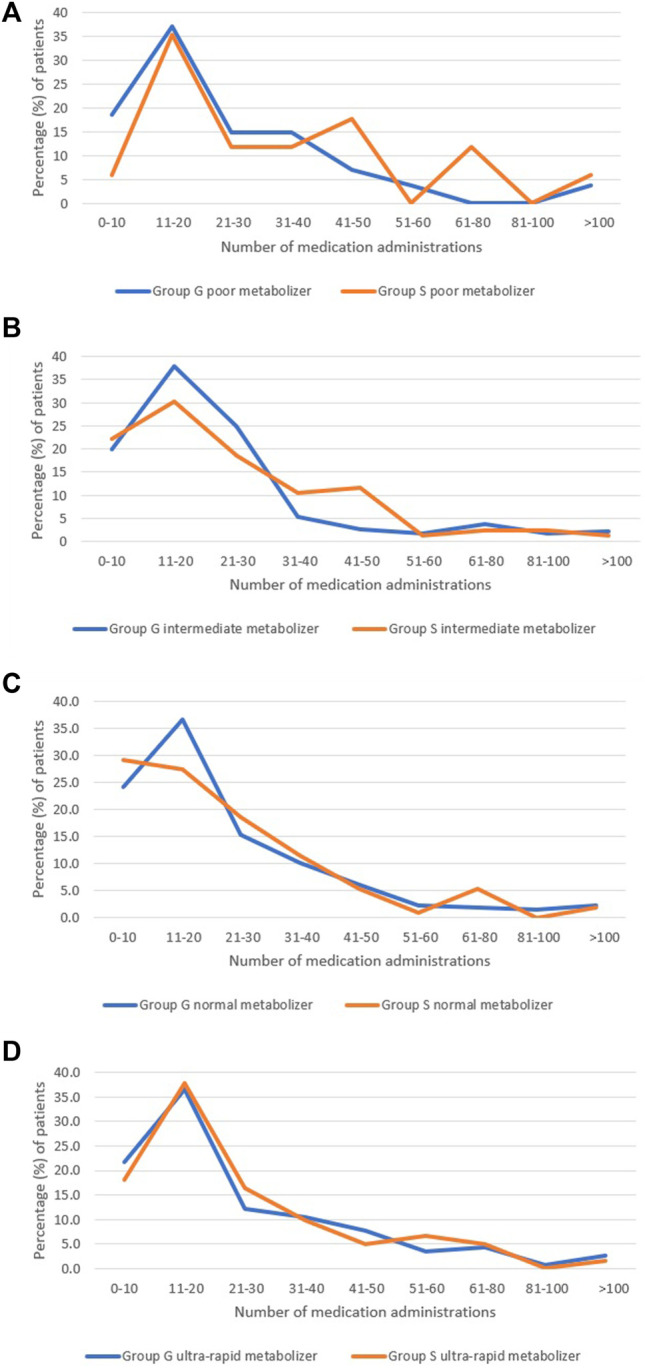
Summary of number of psychotropic medication administrations among Groups G and S stratified by CYP2D6 functional status. **(A)**: Number of psychotropic medication among Groups G and S in poor metabolizers. **(B)**: Number of psychotropic medication among Groups G and S in intermediate metabolizers. **(C)**: Number of psychotropic medication among Groups G and S in normal metabolizers. **(D)**: Number of psychotropic medication among Groups G and S in ultra-rapid metabolizers.

Data analysis for the number of psychotropic medication administrations was restricted to patients with a LOS >3 days and who were treated with major CYP2D6 substrates. The following psychotropic major CYP2D6 substrate medications were administered during this study: haloperidol, mirtazapine, aripiprazole, fluoxetine, risperidone, venlafaxine, duloxetine, clonidine, chlorpromazine, amitriptyline, fluphenazine, perphenazine, doxepin, nortriptyline, fluvoxamine, clomipramine and imipramine. Overall, fewer patients were administered major CYP2D6 substrates in group G (68.1%) compared to S (71.6%). Overall, PMs in group G received less CYP2D6 major substrates compared to group S. No differences were observed for UMs regarding administration of major CYP2D6 substrates between groups G and S. Refer to Figures 2A–D. Overall, Blacks were prescribed less psychotropics compared to Whites and Latinos. Refer to Table 1.

## Discussion

In our evaluation of the CYP-GUIDES data, we discuss the importance of including race/ethnicity in PGx studies and the need for using updated standardized reporting of CYP2D6 phenotypes. These are important pharmacovigilant factors underrepresented in pharmacogenomic clinical studies which can impact therapeutic recommendations and ultimately treatment outcomes in managing MDD.

### Prevalence of CYP2D6 Phenotypes and Drug-Gene Interactions

Patient population from the CYP-GUIDES trial was racially/ethnically diverse with Whites (57.5%), Latinos (25.6%) and Blacks (12.3%). Reported diagnoses varied by race/ethnicity with MDD with recurrence commonly representative as the primary diagnosis.

Most patients were CYP2D6 NMs (40.2%), followed by IMs (34.3%), UMs (20.3%), and PMs (5.1%). PMs were more commonly representative among Whites (6.8%) and UMs among Latinos (24.4%). Overall, prevalence of DGIs for the total study population was approximately 40%. Notably significantly fewer DGIs (24%) occurred in Blacks; this could be secondary to the lower prevalence of PMs and UMs in CYP2D6 in this race and greater prevalence of NM status (54.8%) compared to Whites and Latinos. Furthermore, Blacks also used less CYP2D6 major substrate psychotropic medications (57%) compared to Whites (66%) and Latinos (69.4%).

In our sub-analysis, using a reclassified definition of activity scores of CYP2D6 phenotypes, we observed greater representation of UMs than previously reported in the CYP-GUIDES trial (20.3 versus 6%, respectively) (Tortora et al., 2020). This step of pharmacovigilance demonstrates the importance of using standardized reporting to guide therapeutic recommendations regarding utility of CYP2D6 substrate medications because these can potentially impact treatment outcomes such as LOS and RAR.

### Length of Stay (LOS)

In our sub-analysis we did not observe significant differences in LOS between groups G and S for patients admitted for >3 days using both CEMS and Epic or admitted for >3 days using CEMS only. It is possible that the overall LOS appeared to be longer with regards to the former since physicians were more familiar with CEMS than with Epic introduced later in the study. Furthermore, when stratifying data analyses for >3 days and using CEMS only by CYP2D6 phenotype, PMs in group G had significantly shorter LOS compared to group S (median 5.8 days versus median 11.4 days; *p* = 0.002). Similar trends were noted by a recent sub-analysis by Ruaño et al. (2021), in which they observed LOS to be significantly shorter (mean difference of 2 days) among sub-functional patients in group G compared to S. These findings suggest that using PGx clinical decision support to manage depression can help to reduce overall healthcare expenditures associated with LOS, particularly for CYP2D6 PMs who are at increased risk for adverse effects when receiving treatment with medications metabolized predominantly by CYP2D6 (Maciel et al., 2018; Martin et al., 2019).

On the other hand, both IMs and UMs appeared to have a greater LOS in group G versus S in our sub-analysis. One potential explanation for this finding with UMs was that the original CYP-GUIDES trial did not use the updated standardized classification of CYP2D6 phenotype, accounted for in our sub-analysis. Consequently, it is possible that patients who were categorized as NMs in the original trial were in fact UMs missing out on potential opportunities for therapeutic interventions that could have resulted in a shorter LOS in the group G versus S.

Latinos and Blacks had greater LOS in group G versus S, but the contrary was observed among Whites. Given that there was a greater prevalence of PMs in Whites compared to Blacks and Latinos, it is possible that Whites in group G benefitted the most from PGx guidance administration of psychotropics resulting in a shorter LOS than those in group S. Overall Latinos had a shorter LOS compared to Whites (median difference = 0.8 days). Latinos experience various challenges when seeking care for depression. Some of these challenges include communication barriers, stigma, lack of insurance (Camacho et al., 2015; Sanchez et al., 2019; American Psychiatric Assocation, 2021) which might explain the shorter overall LOS experienced by Latinos in our sub-analysis.

### Readmission Rates (RAR)

In our sub-analysis, race/ethnicity was significantly associated with RAR. In particular, lower RAR was evident among Latinos in group G versus S, but reportedly greater among Blacks and Whites in group G versus S. This could be due to a greater percentage of patients with MDD recurrence diagnosis in group G in Blacks (41.5%) and Whites (48.4%) compared to Latinos (32.5%). Takahasi et al. (2017) reported that patients with supra CYP2D6 functional status were more likely to be readmitted. This finding is in contrast to our sub-analysis which showed lower RAR among Latinos, even though a significant proportion of these individuals were categorized as UMs. However, patients in the Takahasi study were older (median age: 49 years) and followed over a period of 9 years. In another study, Berges. (2015) found that older patients with greater severity of depressive symptoms were at higher risk for re-admission. Kompella et al. (2021) reported that older age, race (Caucasians versus Blacks and Latinos), and medication non-adherence increased the risk of readmission. Our sub-analysis supports similar findings of higher RAR among Whites who were older. Furthermore, a greater proportion of White patients compared to Blacks and Latinos in our sub-analysis had a diagnosis of MDD recurrence at admission, potentially contributing to the higher RAR and LOS observed among Whites.

### Number of Psychotropic Medication Administrations

Majority of the medications used for treatment of depression are metabolized by CYP450 enzymes, specifically, CYP2D6 and CYP2C19 (Brøsen, 2004). CYP2D6 is a highly polymorphic enzyme metabolizing approximately 25% of commonly used medications (Whirl-Carrillo et al., 2021) and up to 80% of psychotropic medications (Panza et al*.*, 2016). A patient’s CYP2D6 phenotype can influence response to treatment with medications which are major CYP2D6 substrates. Our sub-analysis included patients with a LOS >3 days and who were treated with major CYP2D6 substrates. Overall, fewer patients were administered major CYP2D6 substrates in group G compared to group S with this finding particularly reflective of PMs. This is consistent with Ruaño et al. (2021) reporting PGx guided therapy to reduce the use of major CYP2D6 substrates in patients with MDD, especially, for patients who had CYP2D6 sub-functional status. Unlike Ruaño study 2021, our sub-analysis accounted for UMs and used the standardized genotype to phenotype classification for CYP2D6. In our sub-analysis, no differences were observed for UMs related to administration of major CYP2D6 substrates between groups G and S. It is possible that therapeutic guidance, interventions made by prescribers, and treatment outcomes including LOS and RAR may have appeared differently had reclassification of UM status been used previously in the original CYP-GUIDES trial. Latinos and Whites appeared to use more psychotropics compared to Blacks. This may be attributed to potentially greater severity of diagnoses requiring treatment augmentation in these former race/ethnic populations.

### Limitations

Our sub-analysis is not without limitations. The original CYP-GUIDES dataset did not include patient comorbid conditions, other concurrent non-psychotropic medications and socio-economic status (for example-insurance coverage) which could be considered confounding factors influencing treatment outcomes (Tortora et al., 2020; Ghosh et al., 2021). The study was also conducted at only one facility. Although majority of antidepressants are metabolized by CYP2C19 and CYP2D6 (Budhwani et al., 2015), only CYP2D6 genotyping was conducted during the CYP-GUIDES trial. Broader PGx testing information involving CYP2C19 or panel-based testing including additional pharmacokinetic and pharmacodynamic markers from patients in the CYP-GUIDES trial could have provided even more targeted therapeutic guidance to ensure rationale prescribing of psychotropics for management of MDD. Therefore, our analyses cannot be generalized to all patients with depression. Both diagnosis and race/ethnicity were also self-reported by patients. Self-reporting of ethnicity may be a limitation as it may not be a true reflection of the true genetic composition of individuals (Mersha and Abebe, 2015). One potential confounder that we did not account for was phenoconversion. This potential confounder involving drug-drug interactions can ultimately influence the prevalence of DGIs and treatment response. For example, concomitant administration of medications such as bupropion, fluoxetine and paroxetine which are CYP2D6 inhibitors could result in an adjusted CYP2D6 phenotype that is different to genotype-based prediction of drug metabolism (Owen et al., 2009; Cicali et al., 2021; Hahn and Roll, 2021). Another limitation in our sub-analysis includes the small number of patients restricting additional data analyses related to RAR and race/ethnicity (Ruaño et al., 2020). The reader is encouraged to review other operational limitations suggested in the original CYP-GUIDES trial that could be considered for evaluation in future study.

## Conclusion

Using a reclassified definition of AS of CYP2D6 phenotype from genotype, we observed greater representation of UMs than previously reported in the CYP-GUIDES trial, especially, in Latinos. Given the dynamic, budding field of PGx, this step of pharmacovigilance demonstrates the importance of using standardized reporting to guide therapeutic interventions involving psychotropic medications metabolized by CYP2D6. Therapeutic guidance, interventions made by prescribers, and treatment outcomes including LOS and RAR may have reflected differently had a standardized reclassification of UM status been used in the original CYP-GUIDES trial. Future evaluation of the CYP-GUIDES data related to the impact of phenoconversion is warranted.

## Data Availability

Publicly available datasets were analyzed in this study. This data can be found here: https://data.mendeley.com/datasets/25yjwbphn4/1.
